# Increased Feeding and Nutrient Excretion of Adult Antarctic Krill, *Euphausia superba*, Exposed to Enhanced Carbon Dioxide (CO_2_)

**DOI:** 10.1371/journal.pone.0052224

**Published:** 2012-12-26

**Authors:** Grace K. Saba, Oscar Schofield, Joseph J. Torres, Erica H. Ombres, Deborah K. Steinberg

**Affiliations:** 1 Institute of Marine and Coastal Sciences, Rutgers University, New Brunswick, New Jersey, United States of America; 2 College of Marine Science, University of South Florida, St. Petersburg, Florida, United States of America; 3 Virginia Institute of Marine Science, College of William & Mary, Gloucester Point, Virginia, United States of America; University of North Carolina Wilmington, United States of America

## Abstract

Ocean acidification has a wide-ranging potential for impacting the physiology and metabolism of zooplankton. Sufficiently elevated CO_2_ concentrations can alter internal acid-base balance, compromising homeostatic regulation and disrupting internal systems ranging from oxygen transport to ion balance. We assessed feeding and nutrient excretion rates in natural populations of the keystone species *Euphausia superba* (Antarctic krill) by conducting a CO_2_ perturbation experiment at ambient and elevated atmospheric CO_2_ levels in January 2011 along the West Antarctic Peninsula (WAP). Under elevated CO_2_ conditions (∼672 ppm), ingestion rates of krill averaged 78 µg C individual^−1^ d^−1^ and were 3.5 times higher than krill ingestion rates at ambient, present day CO_2_ concentrations. Additionally, rates of ammonium, phosphate, and dissolved organic carbon (DOC) excretion by krill were 1.5, 1.5, and 3.0 times higher, respectively, in the high CO_2_ treatment than at ambient CO_2_ concentrations. Excretion of urea, however, was ∼17% lower in the high CO_2_ treatment, suggesting differences in catabolic processes of krill between treatments. Activities of key metabolic enzymes, malate dehydrogenase (MDH) and lactate dehydrogenase (LDH), were consistently higher in the high CO_2_ treatment. The observed shifts in metabolism are consistent with increased physiological costs associated with regulating internal acid-base equilibria. This represents an additional stress that may hamper growth and reproduction, which would negatively impact an already declining krill population along the WAP.

## Introduction

The Antarctic krill, *Euphausia superba*, is a key species in Antarctic food webs [1], [2]. *E. superba* is a major consumer of phytoplankton [3]–[5] and a primary food source for many of the top predators in the Southern Ocean including baleen whales, seals, penguins, and flighted sea birds [6]. Through their feeding and excretion processes, krill are a major source of regenerated nutrients, which in turn support phytoplankton growth [7]–[9]. However, krill along the West Antarctic Peninsula (WAP) region, the northernmost part of the mainland of Antarctica extending into the Southern Ocean, have declined two-fold since the mid-1970s due to profound changes along the WAP in the past decades [2]. The west coast of the northern WAP is changing from a cold, dry polar climate to a warmer, humid subantarctic climate [10] as a result of a 6°C increase in mid-winter surface atmospheric temperatures (>than 5× the global average) in the past 50 years [11], [12]. This rapid warming is increasing the heat content of seawater over the shelf [13] and reducing both amount and duration of sea ice [14], [15]. Concurrent changes in the biomass and composition of krill food source have occurred [10], [16]. WAP summertime chlorophyll *a* (Chl *a*) has declined by 12% over the past 30 years [10], and, in the northern WAP region, there has been a non-uniform shift in the phytoplankton size fraction from large diatoms to small cells (<20 µm) [10], [17], on which krill can not efficiently feed [18]–[22]. Ocean acidification poses an additional threat to krill populations, as there are predictions that by the end of this century, the Southern Ocean will be the first region to be affected by seawater chemistry changes associated with enhanced carbon dioxide (CO_2_) [23], [24].

Human activities have driven the rapid 40% increase in atmospheric carbon dioxide CO_2_, from preindustrial levels of 280 ppm (parts per million) to current levels of nearly 397 ppm [25], [26]. Present-day atmospheric CO_2_ concentration is projected to double by the end of the 21^st^ century [25], [27]. Nearly one-third of emitted anthropogenic CO_2_ is absorbed by the oceans [28], [29], resulting in reductions in seawater pH and alterations in carbonate chemistry (i.e., reductions in carbonate, CO_3_^2−^, ions). The current rapid rate of oceanic CO_2_ uptake, one million metric tons of CO_2_ per hour [30], is paralleled by rates of acidification at least ten times faster than any change seen in the fossil record over the past 65 million years [31]. These rapid changes are expected to cause adverse ecosystem wide effects [32]–[35].

Not only are Antarctic krill exposed to seasonal fluctuations of seawater *p*CO_2_/pH [24], they can migrate between the surface and at depth during ontogenetic migration (700–1000 m; [36], [37]) and diurnal vertical migration (400+m; [38], [39]). As such they are currently exposed not only to a wide range of seawater *p*CO_2_/pH on short (∼daily) time scales, but also to hypercapnic water at depth [40]. Model projections using the IPCC IS92a scenario demonstrated that Southern Ocean seawater *p*CO_2_, within the depth range utilized by Antarctic krill, could rise up to ∼1400 ppm by the year 2100 [40].

Elevated seawater CO_2_ can impact marine organisms both via decreased carbonate saturation that affects calcification rates and via disturbance to acid-base (metabolic) physiology [23], [41], [42]. Organisms have different responses to hypercapnia, the CO_2_-induced acidification of body fluids. Many studies have demonstrated either no effect of CO_2_ on metabolism of organisms [43] or reduced metabolic activity under elevated CO_2_ [34], [41], [44]–[48]. Some organisms in environments predisposed to high fluctuations in pH and CO_2_ over short time scales, such as coastal upwelling regions, cannot compensate and suppress metabolism when they encounter low pH [34], [41], [44], [45]. Other organisms can fully compensate extracellular fluid pH; however, as a result acid-base and ion equilibria reach new steady state values [45], [49]. The result of this adaptation is that there are extra costs of compensation. For instance, organisms may have higher demands for acid-base regulator proteins (e.g., [50]) and would have to work harder to maintain or alter internal acid-base equilibria. Furthermore, their oxygen transport system may be compromised [41], [51], making them less effective at picking up oxygen (O_2_) and forcing them to process more water to extract the O_2_ they demand. For example, *E. superba* have a pH sensitive respiratory protein that could impair oxygen transport depending on blood buffering [52]. The total amount of oxygen that can be carried in the blood in support of routine activities is reduced by half in *E. superba* under an increase in *p*CO_2_ to 1000 ppm (drop in arterial pH from 8.1 to 7.9) [52]. The two strategies (incomplete or complete compensation) may affect the energetics and performance of an organism differently.

Most studies testing CO_2_/pH effects on crustacean zooplankton thus far have focused on growth, development, or mortality of various life stages [40], [53], [54], and a majority of them show decreased hatching success, irregular larval development, or decrease in larval size under conditions of high CO_2_ (elevated above ambient) or low pH (below ambient). The subtle effects on the physiology and metabolism of marine zooplankton due to ocean acidification are extremely understudied. For example, no studies have directly measured zooplankton feeding, nutrient release, or metabolism under CO_2_ levels predicted for the future ocean. In one study, however, amphipods exposed to high CO_2_ concentrations (low pH), exhibited an increase in the expression of the metabolic enzyme glyceraldehyde-3-phosphate dehydrogenase gene (*gapdh* gene), suggesting that metabolic changes occurred in response to acidification [55]. Increases in metabolic enzyme expression and ventilatory frequency and effort have also been demonstrated for some fish and elasmobranchs [56]–[58] and brittle stars [59]. Such increases may be attributed to enhanced metabolic costs. Compensation costs of enhanced CO_2_ may cause long-term shifts in respiration and metabolic equilibria and eventually hamper growth and reproduction of organisms [41], [42], and may eventually negatively impact an already declining krill population.

We conducted a field CO_2_ perturbation experiment along the WAP during the austral summer (January 2011) to determine krill feeding and nutrient excretion rates at ambient (∼390 ppm) and “high” (750 ppm) atmospheric CO_2_ concentrations. We selected the “high” CO_2_ level of 750 ppm because it represents the mean predicted atmospheric CO_2_ concentration by 2100. The experimental design strategy of selecting ambient and 750 ppm when using two CO_2_ treatments is also what is recommended in the Guide to Best Practices for Ocean Acidification Research and Data Reporting [60]. However, in the present/future oceans krill are/will be exposed to large fluctuations in *p*CO_2_ and pH due to strong deviations of seawater chemistry with atmospheric CO_2_ concentration [61]; thus, the results from this study likely reflect mean responses to enhanced CO_2_. We hypothesized that under conditions of high CO_2_ in perturbation experiments, krill grazing rate and metabolism (nutrient excretion, metabolic enzyme activity) would be increased, reflecting extra costs of compensation due to maintaining internal acid-base balance.

## Materials and Methods

### Ethics statement

All animal work has been conducted according to relevant national and international guidelines. No specific permits were required for the described field studies per articles of The Antarctic Treaty and the National Science Foundation, which is charged with enforcement of the Treaty in the USA and its possessions. The location (Antarctica) is protected under the provisions of The Antarctic treaty, but these provisions do not extend to valid scientific research supported by a recognized national research program of a signatory nation. No endangered or protected species were involved.

### CO_2_ Perturbations

Seawater for sampling the initial phytoplankton composition in the water column was collected at various depths, ∼100 km offshore of Adelaide Island along the West Antarctic Peninsula in January 2011 (66.51°W, 69.87°S) aboard the R/V Laurence M. Gould using Niskin bottles affixed to a conductivity, temperature, depth (CTD) profiler. A known volume of collected seawater from each depth was filtered onto a GF/F filter and flash frozen for HPLC pigment analysis. The taxonomic composition of the phytoplankton assemblages was derived quantitatively from an analysis of HPLC pigment data using CHEMTAX (V195) [62], [63].

Seawater collected at 20 m depth from the CTD cast used for pigment analysis was also used for the experimental incubations. Seawater was gently siphoned from the Niskin bottles via silicon tubing affixed with 200 µm mesh screen caps, to exclude large zooplankton, into thirty-two, 2-liter round, clear, acid-cleaned polycarbonate bottles equipped with custom-made gas inflow and sampling ports. The seawater in 16 bottles was bubbled continuously with ambient air using aquarium pumps, and the seawater in another 16 bottles was bubbled continuously with commercial air/*p*CO_2_ gas mixtures (custom mixed by Linde/Spectra Gases) at the target “high” CO_2_ level of 750 ppm. All bottles were placed in a flow-through seawater tank on the ship’s deck, maintaining a temperature within±1°C of initial ambient sea surface temperature (0.65°C). One layer of neutral density screening was used to reduce light to ∼50% of surface irradiance. The seawater was bubbled with target *p*CO_2_ concentrations for 24 hours (Table 1). During this equilibration period, Antarctic krill were collected via near-surface net tows (2-m square-frame net with 700 µm mesh and a non-filtering cod end) near the same location as the seawater collection. Tows were sorted, and 40 adult *Euphausia superba* of similar size (20 non-gravid, and 20 gravid females) were gently placed into a 30-liter tub filled with filtered seawater. The non-gravid krill were selected solely based on size. Their sex was not determined, so both males and females were likely included in the treatments. The selected krill were allowed to empty their guts (∼12 hours) prior to the experimental incubations. After the 24-hour equilibration, 3 seawater bottles from each the ambient (390 ppm) and high (750 ppm) CO_2_ treatments were sacrificed for a suite of replicate measurements (see below), and served as the initial, or T_0_, time point. Additionally, 10 non-gravid and 10 gravid presorted krill were sacrificed for initial sampling of metabolic enzyme activity and biochemical composition (see below). From the remaining 13 seawater bottles for each CO_2_ treatment, 1 adult non-gravid krill was added to each of 5 bottles (1 krill/bottle), 1 adult gravid female was added to each of 5 bottles (1 krill/bottle), and 3 bottles remained without krill and served as controls. The bottles were incubated and continuously bubbled with target CO_2_ concentrations at a flow rate of 1 ml min^−1^ (∼1 bubble sec^−1^) as described above for an additional 24 hours. Another suite of seawater samples and the remaining krill from the incubation bottles were collected at this final time point, T_f_.

**Table 1:** Carbonate chemistry.

Treatment	n	DIC (µmol kg^−1^)	A_T_ (µmol kg^−1^)	pH	*p*CO_2_ (ppm)	Ω_Ar_
T_0_ Amb	3	2148.9±3.3	2301.9±20.1	8.12±0.06	325.3±43.5	1.694±0.213
T_0_ High	3	2218.5±21.8	2297.8±20.8	7.91±0.07	554.4±92.9	1.098±0.181
T_f_ Amb Control	3	2157.1±12.5	2281.3±4.4	8.05±0.04	389.2±45.5	1.440±0.138
T_f_ Amb+Krill	10	2207.9±13.5	2314.6±14.0	7.99±0.02	452.3±26.0	1.302±0.066
T_f_ High Control	3	2245.6±17.4	2320.3±3.7	7.89±0.06	581.3±98.9	1.071±0.149
T_f_ High+Krill	8	2269.0±11.3	2330.8±25.1	7.84±0.08	671.5±120.6	0.962±0.165

Average (± 1 SD) carbonate chemistry parameters in incubation bottles after a 24-hour acclimation period (T_0_) and after an additional 24-hour incubation (T_f_) in control (no krill added) and treatment (+Krill) bottles bubbled with ambient (Amb) and high concentrations of CO_2_. DIC, dissolved inorganic carbon; A_T_, total alkalinity; Ω_Ar_, aragonite saturation state. Note two of ten samples (n = 8) were lost for the T_f_ High+krill treatment.

### Seawater Sample Collection and Analysis

The seawater samples collected at T_0_ and T_f_ include carbonate chemistry parameters (salinity, dissolved inorganic carbon [DIC], total alkalinity [A_T_], dissolved reactive silicate, phosphate [PO_4_^3−^]); bacterial abundance; phytoplankton and microzooplankton abundance, size, and identification; chlorophyll, particulate carbon and nitrogen, dissolved organic carbon (DOC), urea, ammonium (NH_4_^+^), nitrate (NO_3_^−^), and total dissolved nitrogen (TDN).

Salinity was determined with a Guideline AutoSal salinometer from samples collected from each incubation bottle. BOD bottles (300 ml) for DIC/ A_T_ analysis were allowed to overflow at least one to two volumes and then filled via siphoning directly from each of the incubation bottles. The samples were each fixed with 200 µl of saturated mercuric chloride, sealed with a pre-greased glass stopper followed by tape, and stored in a cool, dark location until analysis. DIC was determined using a coulometer to measure the amount of released CO_2_ after the sample is mixed with phosphoric acid [64]. A_T_ was determined with an open-cell, potentiometric titration of seawater with 0.1 M HCl following the potential of a pH electrode [64]. Peak-area measurements from the DIC and A_T_ analyses were calibrated using certified reference materials (CRMs) obtained from Andrew Dickson at UCSD Scripps Institute of Oceanography. Additionally, an aliquot of seawater from each incubation bottle was siphoned into a 60 ml syringe, filtered through a 0.8 µm syringe filter into a 50 ml polypropylene centrifuge tube, and frozen until later spectrophotometric determination of dissolved reactive silicate [65]. Final carbonate system parameters and pH were calculated using CO2 calc software [66] using a total pH scale (mol/kg-SW), K1 and K2 constants [67] with refits [68], and the acidity constant of KHSO_4_ in seawater [69].

Whole water samples for algal and protozoan cell counts were collected in duplicate from each experimental bottle, one preserved with acid Lugol’s solution (final concentration 5%) and one preserved with 37% buffered formaldehyde. Subsamples for algal identification of major taxa (cryptophytes and large [>20 µm] diatoms) and cell counts were settled in 1 ml Sedgewick rafters, and three replicate frames of at least 100 cells were counted with a Nikon DIAPHOT-TMD inverted microscope at 600X magnification. Subsamples (50–100 ml) for protozoans (ciliates, heterotrophic dinoflagellates) were settled for at least 24 hours in Utermöhl settling chambers, after which the entire contents were counted under an inverted microscope [70], [71]. An aliquot of seawater from each incubation bottle was also filtered onto a GF/F filter, which was wrapped in foil and frozen for fluorometric chlorophyll *a* (chl *a*) analysis [72]. Clearance and ingestion rates of *Euphausia superba* on prey were calculated according to the equations of Frost [73]. Chl *a* ingestion rates were converted to carbon (C) using the C:Chl ratio of 63∶1 determined previously in the study region [74] using the methods of Ducklow et al. [75]. Cell volumes of ciliates were calculated according to geometric shapes with linear measurements made via microscopy (minimum of n = 50 per cell type). Carbon contents for ingestion rate conversions were then estimated using cell C to volume conversions for ciliates from Putt and Stoecker [76].

A known volume of seawater from each incubation bottle was also filtered onto a combusted GF/F filter for particulate carbon and nitrogen analysis using a Carbon-Hydrogen-Nitrogen elemental analyzer (Carlo Erba Instruments, NA 1500 Series 2). The remaining filtrate for each replicate was analyzed for dissolved organic and inorganic nutrient concentrations. Concentration of DOC was measured with a Shimadzu TOC analyzer V (minimum detection limit, MDL = 0.5–1.0 µmol l^−1^; coefficient of variance, CV = 2–644%) after acidification and purging of dissolved inorganic carbon [77], [78]. Ammonium was measured with the phenol/hypochlorite Koroleff method with MDL = 0.05 µmol l^−1^ and CV = 2.5% [72], [79] and urea was measured with the diacetyl monoxime procedure with MDL = 0.05 µmol l^−1^ and CV = 2% [80]. Concentrations of PO_4_^3−^ (Koroleff method) (MDL = 0.05 µmol l^−1^; CV = 2–3%) were determined with a QuikChem 8500 AutoAnalyzer [79].

Krill nutrient release rates (ng individual^−1^ hour^−1^) were calculated according to the following equation:
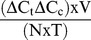
where ΔC_t_ is the change in nutrient concentrations (ng l^−1^ day^−1^) in the treatment bottles and ΔC_c_ is the average change in nutrient concentrations (ng l^−1^ day^−1^) in the control bottles; V is the incubation volume (l), N is the number of grazers in the treatment bottles, and T is incubation time (24 hours day^−1^). Nutrient uptake by algae and bacteria likely occurred during the incubations, and this nutrient decline is incorporated in this equation in the controls as ΔC_c_.

### Metabolic Enzyme Activities and Chemical Composition of Krill

Krill collected at the start and end of the perturbation experiment were placed into individual 5 ml cryovials, flash frozen in liquid nitrogen, and stored at −80 °C until analysis for biochemical composition in the laboratory. A subset of replicates were analyzed for a suite of measurements for metabolic enzyme activities and biochemical composition. Two adult non-gravid krill and two adult gravid female krill for each of the two CO_2_ treatments at T_0_ and T_f_ were thawed, measured (total length), and weighed (wet weight) prior to placement in individual scintillation vials and placed in a 55°C drying oven for 72 hours. Individual krill were re-weighed (dry weight) then homogenized and subsampled (n = 2 per krill) for total particulate C and N and particulate organic carbon and nitrogen (POC and PON, respectively) after vapor phase acidification with concentrated hydrochloric acid on a Carbon-Hydrogen-Nitrogen elemental analyzer (Carlo Erba Instruments, NA 1500 Series 2) according to Hedges and Stern [81]. Particulate carbonates were calculated as the difference between total particulate C and POC [81]. The remaining three adult non-gravid and three gravid krill for each of the two CO_2_ treatments at T_0_ and T_f_ were homogenized and split for analysis of the following: malate dehydrogenase (MDH) and lactate dehydrogenase (LDH) activities [82] and protein content [83].

### Statistical Analysis

Statistical comparisons of the effects of CO_2_ on feeding rates, nutrient release rates, metabolic enzyme activity, and biochemical composition were made by 1-way ANOVA, employing the p = 0.05 level of significance.

### Data Management

Data reported here are available on the Palmer, Antarctica Long Term Ecological Research (PAL LTER) DataZoo website: http://pal.lternet.edu/data/.

## Results

### Carbonate Chemistry

At the start of the incubations with krill, T_0_, the pH and *p*CO_2_ of seawater in ambient bottles averaged 8.12 and 325 ppm, respectively, and the seawater in high CO_2_ bottles had an average pH and *p*CO_2_ of 7.91 and 554 ppm, respectively (Table 1). T_f_ seawater pH and *p*CO_2_ in ambient treatment bottles with krill averaged 7.99 and 452 ppm, respectively, while seawater pH and *p*CO_2_ in T_f_ high CO_2_ treatment bottles with krill averaged 7.84 and 672 ppm, respectively (Table 1). The differences in pH and *p*CO_2_ between ambient and high CO_2_ treatments were significantly different at T_0_ and at T_f_ (one-way ANOVA, p<0.05). Total alkalinity (A_T_) at T_f_ was on average 27 µmol kg^−1^ higher in the seawater treatments with krill added compared to seawater controls with no krill. Additionally, while seawater dissolved inorganic carbon (DIC) in the high CO_2_ treatment was similar between gravid and non-gravid krill, seawater A_T_ was significantly higher (36 µmol kg^−1^) in the gravid krill treatment, which resulted in significantly lower calculated *p*CO_2_ (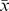 = 563 ppm in gravid, 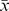 = 779 ppm in non-gravid; p<0.01) and caused high variability in A_T_ and *p*CO_2_ when gravid and non-gravid krill were averaged together (Table 1).

### Composition of Phytoplankton and Microzooplankton

The upper water column at the location where we collected seawater and krill for the experiment was well-mixed and uniform. *In situ* chl *a* biomass in the top 50 m was uniformly low (2.6 µg L^−1^) and then dropped to near undetectable levels below 50 m. Cryptophytes contributed on average 57% (± 1.2% SD) to total phytoplankton chl *a* and thus were the dominant phytoplankton group in our study area (Table 2). Contributions to total phytoplankton chl *a* by other taxa were much lower (diatoms, 35%; mixed flagellates, 4%; prasinophytes, 2%; and type 4 haptophytes, 1%; Table 2). Abundances of ciliates in our experimental bottles were very low (∼2–3 cells ml^−1^; Table 2), and heterotrophic dinoflagellates were not detected in our 100 ml seawater subsamples.

**Table 2:** Initial composition and abundance of phytoplankton and microzooplankton.

HPLC PIGMENT CHEMTAX ANALYSIS (% contribution to Chlorophyll *a*)					
Treatment	Cryptophytes	Diatoms	Mixed flagellates	Prasinophytes	Type 4 Haptophytes
WC_0–50 m_	57.3±1.2	35.3±4.2	4.1±6.5	2.0±1.8	1.3±0.9
**MICROSCOPIC ANALYSIS (cells ml^−1^)**					
**Treatment**	**Cryptophytes<10 µm**	**Diatoms>10 µm**	**Ciliates>10 µm**		
T_0_ Amb	351±42	110±4	2.3±1.0		
T_0_ High	267±44	78±6	2.7±0.1		

Top panel: The relative contribution of each of the five main phytoplankton groups in the WAP region to total chlorophyll *a* in the top 50 m of the water column (WC_0–50 m_) where seawater and krill were collected for the experimental incubations (HPLC pigment CHEMTAX analysis; % contribution to total chlorophyll *a*). Bottom panel: Abundance of dominant phytoplankton (cryptophytes, diatoms) and microzooplankton (ciliates) in incubation bottles after a 24-hour acclimation period (T_0_) in control (no krill added) bottles bubbled with ambient (Amb) and high concentrations of CO_2_ (Microscopic analysis; cells ml^−1^). Values are mean±1 SD.

### Feeding Rates

**Figure 1:**
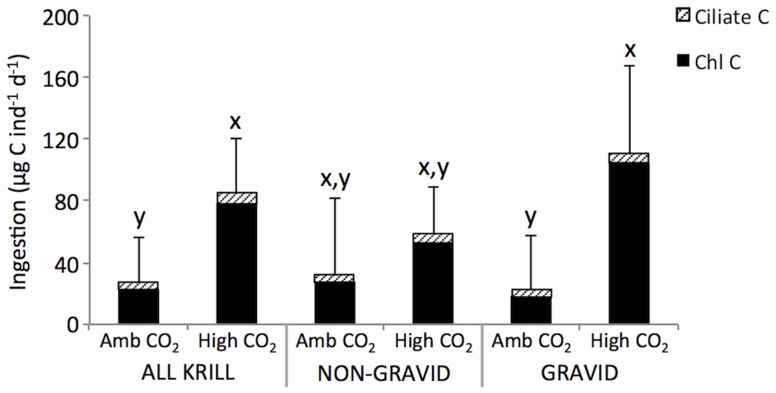
*Euphausia superba* ingestion rates. Chlorophyll *a* and ciliate carbon ingestion rates of krill exposed to ambient (Amb) and high (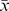 = 672 ppm) concentrations of CO_2_. Chlorophyll *a* was converted to C using a C:chl ratio of 63∶1 estimated in our study area by Bernard et al. [74]. Ingestion rates of krill on ciliates were converted to C using cell C to volume conversions from Putt and Stoecker [76]. Mean of n = 5 for non-gravid and gravid krill and n = 10 for all krill; error bars = 2×standard error. x and y denote a significant difference in total C ingestion rates between treatments (x>y, p<0.05).

Chlorophyll carbon ingestion rates of krill exposed to high CO_2_ averaged 78 µg C individual^−1^ d^−1^ (0.05 % body C d^−1^) and were 3.5 times higher than krill ingestion rates at ambient CO_2_ (p<0.05 for all and gravid krill; Fig. 1). Ingestion rates of ciliates by krill were much lower compared to chlorophyll ingestion rates, averaging 4.7 and 6.3 µg C individual^−1^ d^−1^ in the ambient and high CO_2_ treatments for all krill, respectively (Fig. 1).

### Nutrient Release Rates

**Figure 2:**
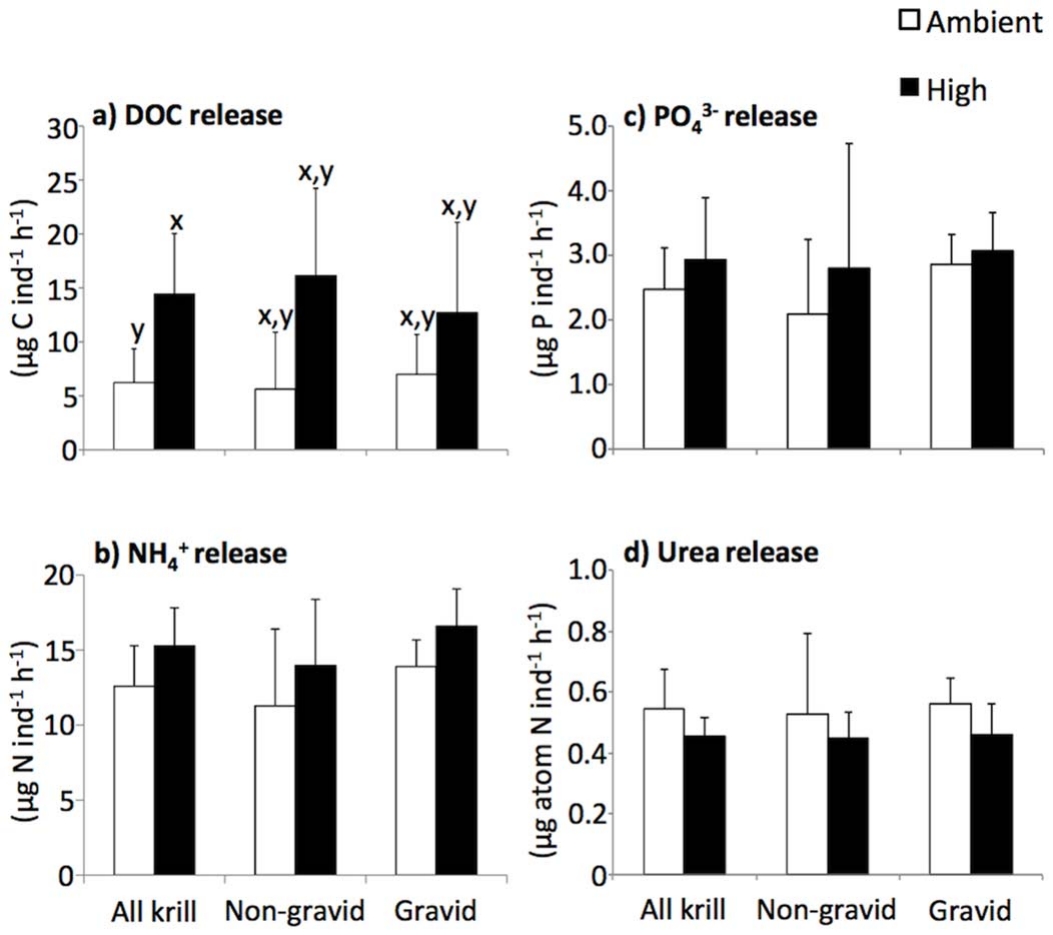
*Euphausia superba* organic and inorganic nutrient release rates. Dissolved organic carbon (DOC; a), ammonium (NH_4_^+^; b), phosphate (PO_4_^3−^; c), and urea (d) release rates of krill exposed to ambient and high (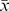 = 672 ppm) concentrations of CO_2_. Mean of n = 5 for non-gravid and gravid krill and n = 10 for all krill; error bars = 2×standard error. For DOC release, x and y denote a significant difference between treatments (x>y, p<0.05).

Release rates of dissolved organic carbon (DOC), ammonium (NH_4_^+^), and phosphate (PO_4_^3−^) by krill were up to 3.0, 1.5, and 1.5 times higher, respectively, in the high CO_2_ treatment compared to ambient CO_2_ (Fig. 2). DOC, NH_4_^+^, and PO_4_^3−^ release averaged 0.2% body C d^−1^, 1.1% body N d^−1^, and 1.9% body P d^−1^ (based on a body N:P molar ratio of 20.4 in adult *Euphausia superba*, [84]), respectively, for krill in the high CO_2_ treatment. Release rates of urea, however, were consistently about 17% lower in the high CO_2_ treatment compared to ambient. This resulted in a lower proportion of urea release (% total measured nitrogen: urea+NH_4_^+^) by krill in the high CO_2_ treatment (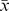 = 29%) compared to ambient (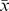 = 38%).

### Krill Chemical Composition and Enzyme Activity

**Figure 3:**
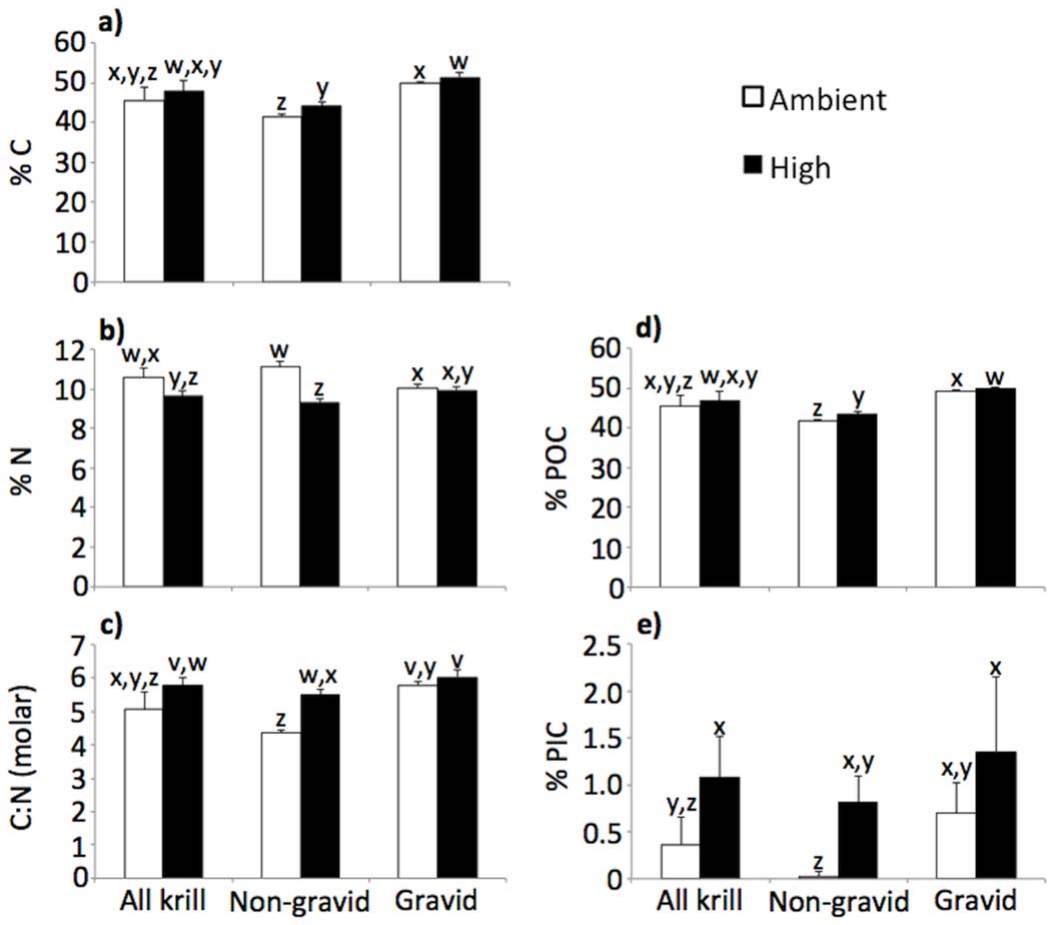
*Euphausia superba* chemical composition. Chemical composition (a: % carbon, %C; b: % nitrogen, %N; c: C:N molar ratio; d: % particulate organic carbon, % POC; and e: % particulate inorganic carbon, % PIC) of krill exposed to ambient and high (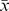 = 672 ppm) concentrations of CO_2_. Mean of n = 4 for non-gravid and gravid krill and n = 8 for all krill; error bars = 2×standard error. v, w, x, and y denote significant differences between treatments (v>w>x>y, p<0.05).

**Figure 4:**
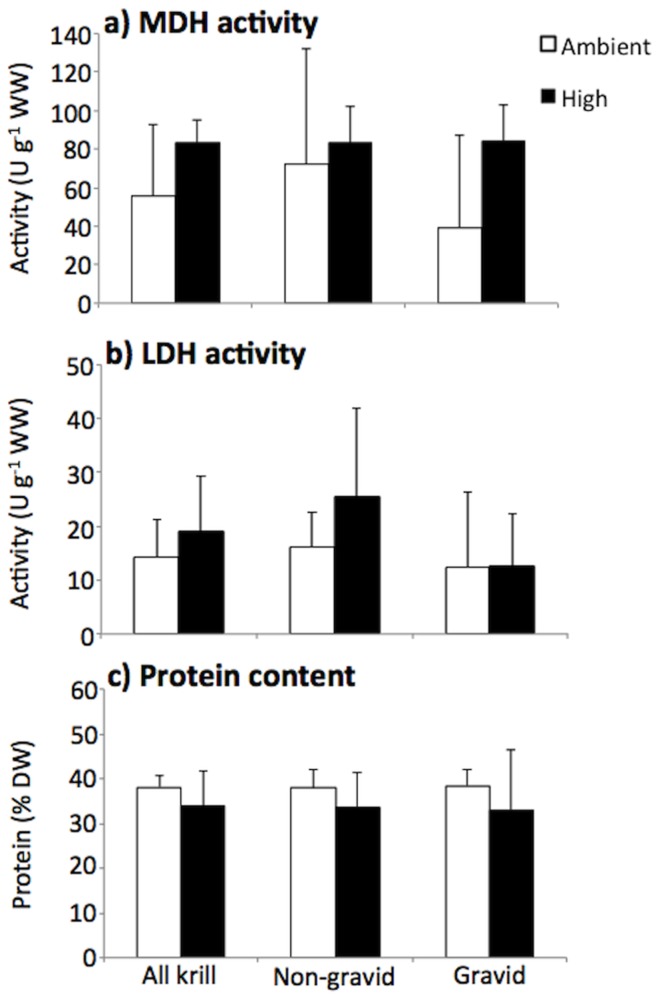
*Euphausia superba* metabolic enzyme activity and protein content. Malate dehydrogenase activity (MDH; a), lactate dehydrogenase activity (LDH; b), and protein (c) of krill exposed to ambient and high (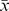 = 672 ppm) concentrations of CO_2_. Mean of n = 3 for non-gravid and gravid krill and n = 6 for all krill; error bars = 2 × standard error.

Total, organic, and inorganic particulate carbon (C) contents (%C, %POC, %PIC) of krill exposed to high CO_2_ were significantly higher than krill incubated in ambient CO_2_ conditions (p<0.05 for %C and %POC in non-gravid and gravid krill and for %PIC in all and non-gravid krill; Fig. 3). Nitrogen (N) contents of krill, however, were lower in the high CO_2_ treatment compared to ambient, and this was most pronounced in non-gravid krill (p<0.05 for all and non-gravid krill; Fig. 3). Higher C and lower N drove higher C:N ratios in krill in the high CO_2_ treatment (p<0.05 for all and non-gravid krill; Fig. 3). Compared to gravid krill, non-gravid krill had lower %C and %POC (p<0.05 for T_0_, T_f_ ambient, and T_f_ high CO_2_), %PIC (p<0.05 for T_0_ and T_f_ ambient), and molar C:N (p<0.05 for T_0_, T_f_ ambient, and T_f_ high CO_2_). However, non-gravid krill had higher %N (p<0.05 for T_0_ and T_f_ ambient) and protein content (Table 3; Fig. 3). The activity of enzymes malate dehydrogenase (MDH) and lactate dehydrogenase (LDH), proxies for overall metabolism and respiration [85]–[87], were consistently higher and protein content was consistently lower in the high CO_2_ treatment compared to ambient (Fig. 4). Additionally, MDH and LDH activities increased from T_0_ to T_f_ in the high CO_2_ treatment and remained unchanged from T_0_ to T_f_ ambient treatment, except for LDH in the T_f_ ambient NG treatment, which increased from T_0_. Protein content of krill, however, increased slightly from T_0_ to T_f_ in the ambient treatment, but decreased from T_0_ to T_f_ in the high CO_2_ treatment. However, there were no significant differences in MDH, LDH, citrate synthase (CS) activity, or protein content between krill type or CO_2_ treatments (p>0.05).

**Table 3:** Physical and chemical characteristics of *Euphausia superba*.

Parameter	Non-gravid	Gravid	All krill
Length (cm)	4.35±0.07	4.85±0.35	4.60±0.36
WW (g)	1.203±0.122	2.236±0.099	1.720±0.603
DW (g)	0.241±0.014	0.477±0.037	0.359±0.138
%C	41.3±1.1	49.7±1.2	45.5±4.6
%N	10.7±0.1	10.0±0.3	10.4±0.4
%POC	41.7±0.4	48.4±1.2	45.0±3.6
C:N (molar)	4.49±0.13	5.79±0.31	5.14±0.73
POC:PON (molar)	4.37 ±0.14	5.62±0.30	4.99±0.70
%PIC	0.23±0.44	1.35±0.89	0.79±0.88
Protein (% DW)	34.7±7.2	31.8±3.3	33.2±5.2
MDH (U g^−1^ WW)	68.9±65.8	35.5±37.7	52.2±37.1
LDH (U g^−1^ WW)	21.8±16.3	9.0±5.0	15.4±9.5

Average (± 1 SD) physical and chemical characteristics of adult non-gravid, gravid, and all (non-gravid and gravid) *Euphausia superba* krill prior to the start of the experiment (T_0_ samples). WW, wet weight; DW, dry weight; C, carbon; N, nitrogen; %POC, percent particulate organic carbon; %PIC, percent particulate inorganic carbon; MDH, malate dehydrogenase and LDH, lactate dehydrogenase activity, in activity (U) per gram WW. Sample size, n, for length and WW was 10 for non-gravid and gravid krill and n = 20 for all krill; n = 2 for DW for non-gravid and gravid krill and n = 4 for all krill; n = 2 for C/N content parameters (%C, %N, %POC, C:N [molar], POC:PON [molar], and %PIC) for non-gravid and gravid krill and n = 4 for all krill (with subsample homogenates [n = 2] analyzed for each krill); n = 3 for protein and MDH for non-gravid and gravid krill and n = 6 for all krill.

## Discussion

Our study is the first to report physiological responses of krill to elevated CO_2_. Previous studies on organismal response to ocean acidification were limited to calcification rates, growth, and the development of early life stages, and studies focusing on zooplankton are scarce. This study demonstrates that *E. superba* respond to elevated CO_2_ by increasing ingestion rate, nutrient release rates, and metabolic activity.

### Carbonate Chemistry

Total alkalinity (A_T_) at the end of the incubation was higher in the seawater treatments with krill added compared to seawater controls with no krill. This was most pronounced in the high CO_2_ gravid krill treatment, contributing to high variability in A_T_ and *p*CO_2_ when calculated for all krill (T_f_ High+krill; Table 1). Higher A_T_ in treatment bottles with krill is likely the result of increased dissolved organic matter (DOM), as evidenced by an accumulation of DOC in these treatments, either through krill excretion or leaching from their egested fecal pellets. The contribution of DOM to A_T_ has previously assumed to be quantitatively insignificant and thus neglected from algorithms used to calculate A_T_. However, recent studies demonstrated that the accumulation of DOC (comprised of weak acids/bases) significantly increased A_T_ [88–[90]. The contribution of dissolved organic nitrogen and phosphorus (DON and DOP, respectively) to A_T_ has not yet been evaluated. Additionally, the magnitude of the contribution of DOM is dependent upon the composition of dissolved organic compounds [89]. DOC release rates (magnitude of DOC accumulation in the experimental bottles) in gravid krill were not significantly different from non-gravid krill, yet A_T_ was higher in the gravid krill treatments. Alternate potential explanations for this discrepancy in A_T_ between gravid and non-gravid krill include: differential composition of dissolved organic compounds released, unequal release of DON and DOP, or differences in fecal pellet production rate or chemical composition yielding differences in surrounding seawater chemistry. These parameters were not examined in our study; thus, the reason for the differences in A_T_ between gravid and non-gravid krill remains unknown but worthy of additional study.

Seawater *p*CO_2_ in the high CO_2_ treatment did not reach target levels of 750 ppm after 48 hours of bubbling (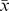 = 581 ppm in T_f_ High control and 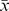 = 672 ppm in T_f_ High+krill; Table 1), suggesting the seawater in the 2L bottles did not fully equilibrate during the course of the incubation. Nonetheless, *p*CO_2_ was significantly higher, and pH and Ω_Ar_ were significantly lower, in the high CO_2_ treatment compared to ambient, and there were significant differential responses in krill feeding, nutrient release, and chemical composition.

### Feeding Rates

Ingestion rates of adult *E. superba* presented here were on the low end of those measured in previous studies. Ingestion rates in our study ranged from 23–99 µg C krill^−1^ d^−1^, which is equivalent to 0.01–0.07 µg chl ind^−1^ h^−1^, 0.005–0.03 µg chl g ww^−1^ h^−1^, and 0.01–0.07% body C d^−1^. These ingestion rates are within range of those found for *E. superba* in the fall (0.01 µg chl ind^−1^ h^−1^, [91]; near 0 µg chl g ww^−1^ h^−1^, [92]) and summer (50–445 µg C krill^−1^ d^−1^; [3]), but lower than others report for *E. superba* in the summer (129–447 µg C krill^−1^ d^−1^; [74]). Clearance rates of *E. superba* in our study (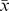 = 8.1 and 10.3 ml krill^−1^ h^−1^ for non-gravid and gravid krill, respectively) were 1–3 orders of magnitude lower than those determined for *E. superba* by Price et al. ([93]; 100–1400 ml krill^−1^ h^−1^). A possible reason for reduced feeding activities of krill in our study is container size, which has been shown to affect clearance rates in krill in a previous study [93]. Clearance rates of *E. superba* were an order of magnitude higher in 50 L tubs [93] than those in 5 L [93], 4.2 L [19], and 2 L [94], [95] bottles. This response has been attributed to stress, abnormal swimming behavior [93], and insufficient food concentrations causing the “refiltration” of already cleared seawater [96] in small containers. However, our measured clearance rates were 1–2 orders of magnitude lower than previous studies with krill in similarly sized 2 L bottles (134 ml krill^−1^ h^−1^, [94]; 217 ml krill^−1^ h^−1^, [95]); thus, we suggest reduced feeding activity of krill in the present study was due to low available food concentration and phytoplankton composition, specifically the dominance of small cryptophytes.

The minimum carbon ingestion rate required by *E. superba* to meet minimal respiratory costs (MCU) and the minimum particulate carbon concentration in seawater needed to meet respiratory costs (Cm) can be estimated from these equations by Holm-Hansen and Huntley [97]: MCU (µg C krill^−1^ h^−1^) = 0.452W^0.975^, where W is krill dry weight in mg; and Cm (mg C L^−1^) = (MCU*RQ)/(a*F), where RQ is the respiratory quotient (0.8; [93], [98]), a is assimilation efficiency (0.8; [93], [98]), and F is filtration, or clearance rate (ml krill^−1^ h^−1^). Using measured krill dry weights (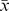 = 241 mg, non-gravid; 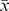 = 477 mg, gravid) and clearance rates (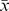 = 8.1 ml krill^−1^ h^−1^, non-gravid; 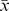 = 10.3 ml krill^−1^ h^−1^, gravid), MCU for non-gravid and gravid krill in our study is 95 and 185 µg C krill^−1^ h^−1^, respectively, and Cm for non-gravid and gravid krill in our study is 12 and 18 mg C L^−1^, respectively. However, the maximum C ingestion rate obtained by krill in our study (gravid, high CO_2_) was only 4.7 µg C krill^−1^ h^−1^, and particulate C concentrations measured at T_0_ prior to krill being added to the bottles averaged only 0.2 mg C L^−1^. Both parameters are 1–2 orders of magnitude lower than that required to meet minimal respiratory costs, and thus were essentially starved during the 24-hour incubation. Reduced feeding activity of krill in the present study was most likely due to a combination of low available food concentration and dominance of cryptophytes. Adult *E. superba* can feed efficiently on microzooplankton (ciliates, heterotrophic dinoflagellates), copepods, and large (>20 µm) phytoplankton (i.e., diatoms) [19], [93], [99]–[101]. Abundances of ciliates in our experimental bottles were very low (∼2–3 cells ml^−1^; Table 2), heterotrophic dinoflagellates were not detected in our 100 ml seawater subsamples, copepods were removed from the seawater with 200 µm mesh screen prior to the experiment, *in situ* chl *a* biomass in the top 50 m where we collected water and krill for the experiment was low (2.6 µg L^−1^), and cryptophytes contributed ∼57% to total chlorophyll (Table 2). Because of their small size (<10 µm), cryptophytes are not efficiently grazed on by krill [18]–[22]. Additionally, the presence of cryptophytes can inhibit krill grazing, as shown for *E. superba* in cryptophyte-dominated assemblages [22]. Thus, it is possible that krill were not actively feeding and were starving *in situ* before we collected them for the incubation.

We also consider potential direct effects of bubbling on feeding processes of krill in our study. No direct comparison of krill feeding rates in aerated vs. non-aerated bottles has been conducted; thus, there is no scientific evidence to support or reject any claims that bubbling caused low feeding rates of krill in our study. Although our estimated grazing rates for krill were low, they were still within range of those estimated in previous studies [3], [91], [92], in which bottles were not aerated during experimental incubations. Additionally, in a study conducted with copepods feeding on phytoplankton [102], aeration had no apparent direct affect on algae consumption. Moreover, *Euphausia pacifica* krill remained active while feeding in 1-gallon glass jars with gentle aeration for over two months in a study conducted by Yen et al. [103], and *E. superba* maintained in aerated buckets remained healthy and survived for a longer time compared to krill in non-aerated buckets at the same stock density [104]. Thus, any negative impacts of bubbling on krill feeding rates in our study were likely insignificant compared to impacts of low food availability and the dominance of cryptophytes.

Despite low feeding rates in krill during the incubation, there was a significant CO_2_−dependent response in krill ingestion rates. Ingestion rates in krill were higher in the high CO_2_ treatment compared to ambient. We hypothesize that increased feeding at high CO_2_ reflects the increased energetic cost of maintaining internal acid-base and ionic equilibria. These extra costs of compensation could include a higher demand for acid-base regulator proteins, which was demonstrated in juvenile cephalopods under short-term exposures to elevated CO_2_ [50]. Acid-base compensation under elevated CO_2_ may compromise the oxygen transport system in krill [52]. This will require the organism to process more water to extract the oxygen they demand [41], [51], likely increasing muscular activity and difficulty of feeding. The impacts of this response are reflected not only in krill ingestion rates, but also in krill nutrient release rates and chemical composition.

### Nutrient Release Rates

Higher rates of DOC, NH_4_^+^, and PO_4_^+^release in krill in the high CO_2_ treatments was likely the direct result of higher ingestion rates by krill as shown for zooplankton in previous studies [105], [106]. Release rates of DOC by krill, to our knowledge have only been measured in one other study conducted by Ruiz-Halpern et al. [107]. Their rates of *E. superba* DOC release (ca. 80–202 µmol C g DW^−1^ h^−1^) were higher than those reported here (1.9–2.8 umol C g DW^−1^ h^−1^); however, they conducted their experiments immediately after collection (and likely feeding) and their incubations were short term (5–240 minutes compared to our 24 h incubation). DOC and nutrient release rates decline rapidly with time when zooplankton are incubated in filtered seawater (not feeding), and this was evident by the reduction of DOC release rates from 202 µmol C g DW^−1^ h^−1^ in a 15 min. incubation to 80 µmol C g DW^−1^ h^−1^ in a 240 min. incubation [107]. Ammonium (NH_4_^+^) release rates of adult *E. superba* presented here (4.3–24.3 µg N krill^−1^ h^−1^; 0.7–1.5% body N d^−1^; 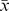 = 12.6 and 15.3 µg N krill^−1^ h^−1^ for krill in ambient and high CO_2_ conditions, respectively) were higher compared to those measured in most previous studies (0.3–3.7 µg N krill^−1^ h^−1^, [108]; 0.6–1.3 µg N krill^−1^ h^−1^, [109];<0.5% body N d^−1^, [110]), but within range of those determined by Ikeda and Mitchell ([111]; 1.2–10.3 µg N krill^−1^ h^−1^, 0.7–1.0% body N d^−1^). Phosphate release rates of *E. superba* in our study (1.0–6.4 µg P krill^−1^ h^−1^; 1.3–2.4 % body P d^−1^; 0.006–0.009 µg P mg DW^−1^ h^−1^; 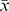 = 2.5 and 3.0 µg P krill^−1^ h^−1^ for krill in ambient and high CO_2_ conditions, respectively) were generally higher than or within range of than those measured for *E. superba* in the summer by Ikeda and Hing Fay ([109]; 0.3–1.6 µg P krill^−1^ h^−1^) and Ishii et al. ([84]; 3.2 µg P krill^−1^ h^−1^), but lower than those measured for krill feeding on copepods in the winter (0.026 µg P mg DW^−1^ h^−1^; [110]). Krill feeding carnivorously tend to have low atomic N:P release ratios (2.02; [110]), whereas starved krill or krill feeding on phytoplankton in the summer have higher N:P release ratios [110], [111]. The atomic N:P release ratio determined in our study averaged 12.9 and 18.4 when calculated for NH_4_^+^and NH_4_^+^+urea, respectively, which is similar to the N:P ratio of 15.3 determined for starved krill in Ikeda and Mitchell [111].

Release rates of urea have not been reported previously for Antarctic krill. The proportion of urea release to total measured nitrogen (20.7–49.5%) were much higher than those found for a tropical euphausiid (1% of total N excreted; [112]). Although direct urea release was not determined in *E. superba* by Ruiz-Halpern et al. [107], dissolved organic N (DON) release (difference between measured total N and NH_4_^+^excretion rates), which would include urea, was about 52% of total measured nitrogen in their study. NH_4_^+^is generally the main nitrogenous excretory product of zooplankton [113]. However, urea or DON can be a significant proportion of total N released by zooplankton [114]–[118]. While NH_4_^+^release rates were consistently higher in krill in the high CO_2_ treatment, urea release rates in this treatment were consistently about 17% lower compared to ambient, suggesting potential differences in catabolic processes of krill between treatments. Due to the hypothesized increase in energetic costs under elevated CO_2_, krill in the high CO_2_ treatment were not only ingesting more food but were also metabolizing more N-rich protein (reflected in slightly lower protein contents of krill in the high CO_2_ treatment at T_f_; Fig. 4), which could have led to differences in the release of N byproducts.

### Krill Chemical Composition and Enzyme Activity

Carbon, nitrogen, protein, LDH, and MDH contents determined for *E. superba* in our study were comparable to those found in previous studies [84], [87], [109], [119]. Significant differences in krill carbon content (%C, %POC, and %PIC), like nutrient excretion, may have resulted from different ingestion rates whereby higher ingestion rates increased carbon content. The larger size and expanded thoracic cavity of gravid krill, and the presence of eggs, likely resulted in the higher %C, %POC, %PIC (from the carbonate-containing exoskeleton), and C:N compared to non-gravid krill. Additionally, the non-gravid treatment potentially included both male and female krill. Male krill contain fewer lipids and cholesterol compared to female krill [120], and likely have fewer energetic requirements. Relative feeding rates between krill sexes are unknown; however, we expect them to be lower in males compared to females as demonstrated for *Acartia tonsa* and *Centropages hamatus* copepods [121]–[123].

Higher %PIC of all krill in the high CO_2_ treatment compared to ambient as well as the increase of %PIC of non-gravid krill from T_0_ to T_f_ in the high CO_2_ treatment provides evidence that calcification, or the mineralization of exoskeletal material, is not prohibited at elevated CO_2_ concentration and may in fact be enhanced as previously shown for calcification in brittle stars [59]. Slight, yet consistent, higher activities of MDH and LDH in krill exposed to elevated CO_2_ resulted from increased energetic demands in this treatment. Similarly, enhanced MDH activities were found in the Mediterranean fish *Sparus aurata* under low pH conditions [124].

If changes in krill chemical composition were caused only by differences in ingestion rates, then nitrogen content (%N) of krill would also increase with ingestion rate in the high CO_2_ treatment. However, krill nitrogen contents were lower (driving C:N higher) in the high CO_2_ treatment. This suggests that the compensation for higher energetic demand for acid-base regulation in krill exposed to elevated CO_2_ not only increases metabolic activity (significant increases in feeding and nutrient release; slight, yet consistent increases in enzyme activity), but also creates stoichiometric changes within the krill caused by differential partitioning of C and N and the utilization and catabolism of proteins (reducing body N, increasing N excretion).

### Conclusions

Our results suggest that *Euphausia superba* respond to elevated CO_2_ by increasing ingestion rates, nutrient release rates, and metabolic activity, reflecting enhanced energetic requirements, but at what cost? Increased growth and metabolism was also observed in brittle stars at elevated CO_2_, but the cost - muscle wastage - was substantial [59]. Stoichiometric changes in krill caused by a decrease in %N and increases in %C, %PIC, and C:N as well as shifts in krill N excretory products at elevated CO_2_ could be indications of biochemical changes that we were unable to determine during this limited short-term field study. Subtle ocean-acidification induced shifts in physiological processes could affect growth and reproduction and accelerate population declines. Krill compensating for higher energy requirements at high CO_2_ will increase feeding and nutrient release rates, which, under favorable food conditions, may provide sufficient energy to maintain growth and reproduction. However, in time periods (i.e., winter) or locations (i.e., northern WAP) with lower food availability, and more importantly with continued reductions in phytoplankton biomass as a result of rapid climate change along the WAP [10], [125], krill may not be able to sustain increased energetic costs. Additionally, females may be less tolerant to these future changes compared to male krill due to their relatively higher energetic requirements. Rapid warming in the WAP region will intensify this response, as metabolic rates increase with temperature [126], suggesting that the combined effects of ocean warming and ocean acidification (enhanced energetic costs, decreased oxygen transport) will be detrimental to Antarctic krill. We do not yet know the response of Antarctic krill exposed to chronic elevated CO_2_ or whether or not krill have the capacity to fully compensate under elevated CO_2_. Future ocean acidification studies with Antarctic krill should focus on prolonged exposures, which will be necessary to pinpoint the underlying physiological responses to increase CO_2_, determine potential adaptive strategies of krill to high CO_2_, and to understand the associated feedbacks on the food web and biogeochemical cycles.
